# Maritime sector at verge of change: learning and competence needs in Finnish maritime cluster

**DOI:** 10.1007/s13437-021-00228-0

**Published:** 2021-02-16

**Authors:** Vesa Kilpi, Tomi Solakivi, Tuomas Kiiski

**Affiliations:** Operations and Supply Chain Management, Turku School of Economics at the University of Turku, University of Turku, FI-20014 Turku, Finland

**Keywords:** Maritime logistics, Shipbuilding, Competence gap, Survey, Environment, Automation

## Abstract

Shipping plays an important role in the world, transporting over 80% of international trade and employing over 1.5 million seafarers. The maritime industry, including shipbuilding and equipment manufacturing, is extensive. Both of these interconnected businesses are facing rapid change caused by increasingly speedy technological development and the tightening of environmental regulation. This survey-based research analyzes the current and future competence needs of firms operating in maritime logistics and the maritime industry. The findings indicate that in both contexts, the increasing importance of various general competences is understood and the need is recognized in particular to improve those related to environmental regulation as well as technology and automation. Overall, the gap between current and desired levels of competence is expected to widen. In terms of education, this is likely to affect vocational training and university-level learning differently in that functional competences are emphasized more in the former and social and meta-competences in the latter.

## Introduction

Requirements for industrial skills are changing. Current skills in demand in supply chains focus on interpersonal and professional knowledge, whereas future needs relate to sustainability and automation (Bals et al. 2019). Changes in the business environment underline the need for lifelong learning (Kotzab et al. 2018).

Christopher and Holweg (2011) demonstrate how uncertainties have increased in global supply chains: it is no longer enough to respond to a single incident (however shocking), as broader structural flexibility is expected. The authors conclude that competitive advantages are short-lived in a global economy, and supply chains must adapt to turbulence.

There is a similar change process in the maritime sector (Fjeld et al. 2018; Johns 2018; World Maritime University 2019). Disruptive development (Christensen 2006) of maritime business calls for new and innovative solutions. On one hand, the environmental regulations (e.g., SOx, CO2, and EEDI) of shipping are becoming stricter. On the other hand, in addition to the increasing transport volumes, logistics processes are being automated. Digitalization entails rearranging the division of labor, whereas task automation makes some jobs obsolete (Bessen 2016).

New technology and automation are affecting multiple tasks in maritime work, thereby generating the need for new skills. As routine and repetitive tasks are being automated, tasks that remain require higher order cognitive skills (Bessen 2016; Green 2012). The World Maritime University (2019) recently estimated that automation in maritime transportation will reduce the number of low-skilled jobs during the coming years, whereas high-skilled positions will remain. At the same time, automation will increase skill requirements even in entry-level positions (Klumpp 2018).

One measure of an organization’s capability to learn is its absorptive capacity (Cohen and Levinthal 1990; Flatten et al. 2011). Technology does not provide sustainable advantage if the organization lacks the relevant absorptive capacity, in other words the capability to learn and renew (Francalanci and Morabito 2008). Organizations must be able to explore different solutions to market changes and to adapt promptly. At the same time, they should exploit current capabilities to align processes internally and therefore get the best out of their current resources (Birkinshaw and Gibson 2004).

As environmental awareness increases and technological advancement changes the rules of the industry, we pose the following research question: how does institutional pressure change in the business environment affect the competence requirements of the maritime cluster?

For the pressures, we follow the logic of DiMaggio and Powell (1983) that the forces towards isomorphism originate from three pressures. The regulatory pressures come from political influence through rules and regulations by powerful stakeholders on which the actor depends. As an example of such stakeholders, one could consider the IMO. However, one should note that in this research we are not directly evaluating IMO competence standards such as STCW. Normative pressures originate from non-governmental organizations, customers, and suppliers in the supply chain. Mimetic pressures usually originate from imitation of other, successful peers.

Our aim here is thus to explore how the maritime industry and logistics estimate the general skills requirements to evolve, presumably partly influenced by the before-mentioned pressures. To do this, we analyze survey data collected from maritime logistics firms, such as shipping companies and port operators, and those engaged in the maritime industry, such as shipyards and equipment manufacturers. We compare gaps, first between the current and desired level of competences, and second between their current and future importance. Together these two gaps will indicate the relative significance of the skills that are needed to overcome the coercive pressure of business change. The paper is structured as follows. Section 2 describes the fundamental aspects of change in the maritime cluster. Section 3 presents the key literature on competence development, together with the research propositions. We discuss the research methodology in more detail in Section 4, present the results of the analysis in Section 5, and finally summarize the results and discuss the conclusions in Section 6.

## The evolving landscape of maritime competence

Maritime transport is the backbone of global trade, around 11 billion tonnes being carried in 2018 (UNCTAD 2019). The cargoes are shipped in over 90,000 vessels that constitute an environment in which over 1.6 million seafarers work (ICS 2020).

Given its global nature, shipping is very susceptible to macroeconomic trends and influences. The most recent was the outbreak of COVID-19, which, among other things, caused interruptions in ordinary ship and crew rotations. Physical dimensions and equipment on board are affected by environmental regulations and tightened competition. General trends including increasing the size of vessels and the level of automation have reduced crew numbers in relative terms. Having fewer people onboard increases the workload and broadens the roles (Caesar et al. 2020) of those who remain. Trading patterns are also changing as steps are being taken to reduce the number and duration of port calls—to increase efficiency. All these developments underline the need for flexibility and social skills among present-day seafarers. Crews are notably multi-national, the major suppliers being China and the Philippines (ICS 2020). Managing everyday operations in such an international environment requires constantly increasing levels of language and communication skills.

The competence levels to ensure safety standards affecting seafarers are set by international conventions (e.g., Mindykowski 2017), predominantly the International Convention on Standards of Training, Certification and Watchkeeping for Seafarers (STCW). This process is not expected to change in the future. Regulatory work in shipping is notably rigid, traditionally driven by major accidents in the field. The impact of digitalization has already raised new regulatory issues to be dealt with. Enhancing personnel awareness and practices have proven effective ways of dealing with cybersecurity issues.

Problems related to crew retention, and the consequent potential shortage of seafarers, is well-documented in the literature (Thai et al. 2013). There has also been an ongoing shift among ships’ officers to take landside jobs due to the preferential recruitment of sea-experienced personnel for onshore positions, and for more socially oriented reasons such as separation from family and home (Gardner et al. 2001; Caesar et al. 2020). Other problems relate to unsatisfactory support, poor human resource management, and limited career opportunities (Dinwoodie 2000; Gekara 2009; Thai et al. 2013).

Digitalization and other overarching technological feats are expected to change crew requirements and characteristics. Not surprisingly, awareness of sustainability and, in particular, the need for technological skills are expected to increase in tandem with the level of automation (Cicek et al. 2019). One potential effect of having more shore-based jobs would be to increase job opportunities for women in shipping professions (UNCTAD 2019).

The envisioned growth and rapid technological developments in maritime transportation will create a need for a newly skilled, competent and motivated workforce. Additionally, increasing digitalization and automation in the shipping industry will require a workforce with more diverse and technically advanced knowledge and expertise than many current employees possess (Cicek et al. 2019).

## The theoretical framework and the propositions

### Competence and skills in maritime education

The research literature approaches maritime education from different perspectives, covering a variety of themes. The IAMU's Body of Knowledge for Maritime Professionals (2019) leans on Bloom´s taxonomy in defining the knowledge, skills and attitudes necessary for a career at sea, whereas other studies focus on a specific skill such as maritime English as a requirement for safety and communication (James et al. 2018).

Manuel (2017) defines three levels of competence in maritime education and training, namely, support, operations, and management, but also concludes that the trend is to move away from traditional practice-oriented task-specific training towards linking vocational education with its specific and restricted competence outcomes with more general or deeper components leading to an academic qualification. The author further notes that university-level education in particular is both the source and the product of change.

Paine-Clemes (2006) discusses maritime education and training from the perspective of quality rather than the variety of skills, concluding that a high-quality education is an ongoing process towards a moving target, rather than an easily defined list of competences.

Ng et al. (2011) focus on the increasing role of postgraduate education in the evolution of the maritime business. Earlier Ng et al. (2009) studied the motivation of postgraduate students in maritime education (see also Lau and Ng 2015). Ferritto (2016) makes an interesting contribution to the literature on maritime education in studying the effects of so-called regimental responsibilities on the learning of those working for a US Coast Guard license.

### Need for new competences

Industrial clusters are autonomous, complex, and loose networks of interrelated organizations and institutions sharing some common needs, competences, or capacities that attract suppliers and other resources (Delgado et al. 2014; Rosenfeld 2005). The functions and activities of these clusters are driven by social norms and reciprocity rather than contractual business targets (Rosenfeld 2005). A cluster therefore gives an environmental and institutional structure to competence development and the building of a knowledge base. This, in turn, requires “institutional thickness” in the form of a common vision on how to develop a shared resource base and in which direction (Beer and Meethan 2007).

Institutional theory provides a framework within which to study changes in the business environment such as digital transformation (Hinings et al. 2018). The influence of societal change (e.g., technological progress and environmental awareness) is initially manifest on the organizational level in the form of actions, which leads to the revision of institutional logic on the industry level. Over time, developments on the societal level and actions taken on the organizational level are scripted in new sets of industry-level rules and revised institutional logic (Wright and Zammuto 2013).

DiMaggio and Powell (1983) have identified three mechanisms explaining how organizations respond to uncertainty in an isomorphic manner—ultimately reducing differentiation. Organizations follow coercive rules similarly, and secondly they implement mimetic actions when following others, or thirdly they follow similar normative standards for professionalism. Those who adapt first are likely to gain competitive advantage, which external regulations could constrain at the same time (Zucker 1987).

According to Thornton and Ocasio (2012), an industrial—in this case maritime cluster (cf. Djoumessi et al. 2019)—could establish an institutional logic among industry members. They share a common identity and a common vision providing a sense of order and legitimacy of action. Pressure generated by the surrounding business environment challenges established institutionalized organizational structures.

With regard to organizations, institutional logic explains how they are affected by rules, norms, and habits generated either externally or internally. Accordingly, they may develop technical solutions under coercive pressure (Zucker 1987).

P1: As a cluster, maritime logistics and the maritime industry share a common view on competence.

Efficiency is not a sole driver for the change, but in addition institutional logic explains how organizations are implementing norms and practices (Thornton and Ocasio 2012). However, it is challenging to urge change and replace established organizational routines, which are deeply rooted in practices. New processes—norms, routines, and rules—need to be institutionalized to make the new ways of working real. Given that organizations are interconnected, institutionalized isomorphic structures are tested by other distinct logics (Thornton and Ocasio 2012).

Clusters do not operate in isolation but rather interact with other industries in the region. Knowledge and competence spillovers benefit related industries in the cluster, but at the same time, there is constant competition for scarce resources. Specialization, which is at the core of an industrial cluster, may lead to a shortage of resources and thus to declining growth (Delgado et al. 2014; Laaksonen and Mäkinen 2013).

Notwithstanding the internal inertia of clusters, a complex, adaptive organization works best in volatile and unpredictable environments (Makkonen et al. 2013). Shared values and purposes among cluster members lead to the initiation of a process of autonomous organization. To facilitate change, there needs to be a gap between perceptions and expectations of the current state. However, if the gap is too narrow, change may be considered unnecessary, whereas too wide a gap may be too formidable.

As Zucker (1987) points out, the institutional environment extends requirements beyond technical needs. At the same time, inefficiency may result when there is a need to comply with other than business requirements, such as if an organization adapts to institutional elements coming from outside without aligning and questioning.

P2: Institutional pressure will widen the gap in environmental technology skills in maritime clusters.

P3: Institutional pressure will widen the gap in automation technology skills in maritime clusters.

Doloreux (2006) underlines the importance of reciprocity in interactions, radical innovations, and the capturing of market potential. Pinto et al. (2015) found that participation in innovation activities within the cluster improved quality and broadened the product range at the same time. Makkonen et al. (2013), in turn, discuss the importance of innovativeness in the Finnish maritime industry, noting that the preference is for incremental improvements rather than radical changes. In general, industrial clusters are not at their best in cases of radical innovation (Hammervoll et al. 2014).

Competences have been operationalized within different structures. LeDeist and Winterton’s (2005) holistic model consists of cognitive, functional, social, and meta-dimensions. The cognitive dimension includes conceptual knowledge and understanding; the functional dimension comprises the operational and practical skills needed at work; and social competences include individual attitudes and behavior. Meta-competence is the capability to combine and facilitate learning in the other three competence areas. An example of such a meta-competence connecting the cognitive, social, and technical areas is an innovation process involving the generation, acquisition, integration, and dissemination of knowledge (Piening and Salge 2015).

Tatham et al. (2017) refer to skills rather than competences and categorize those as problem solving (PSS), general management (GMS), interpersonal (IPS), and functional (FS). They conclude that, in the quest for competitive advantage, PSS skills are needed to sense, and GMS and IPS skills to seize opportunities. Finally, functional skills are required to maintain the advantage.

Kotzab et al. (2018) analyzed the skills required in supply chain management and highlighted five underlying themes: analytical, basic functional, occupation-specific, social, and learning. Their results show the increasing importance of continuous (lifelong) learning both in cognitive and in functional competence areas. Tassabehji and Moorhouse (2008) provide a framework for the procurement part of the supply chain: competent performance in complex supply chains requires a broad set of knowledge and skills, and the right attitude.

Among the capabilities required to adapt to environmental changes is that of developing a shared vision and a changing perception of the current state—via contradiction and questioning (Dooley 1997). As Moller (2010) argues, the organization must have capabilities related to sense-making and agenda construction. Competences are acquired and accumulated in a continuous learning process. At its simplest, learning involves copying and repeating rules and routines. However, according to the theory of expansive learning, the circle may expand, and new knowledge is generated by questioning and sensing the conflict between perceived and expected results (Engeström 2007).

P4: As a cluster, maritime logistics and the maritime industry share a common vision of future gaps in competence requirements (e.g. continuous learning).

P5 In the future, the gap between cognitive and meta-competences will be wider than that between functional and operational competences.

## Methodology

We collected our data on competence development needs via an online survey conducted during February–April 2019. The survey was addressed to representatives of organizations operating in the maritime industry, maritime logistics, and research and to educational institutes and authorities focusing on the maritime sector. The survey comprised questions on the importance and required levels of competences currently and in the future. The analysis reported here was limited to organizations operating in maritime logistics and the maritime industry.

An invitation and a link to the survey were sent to 1500 e-mail addresses belonging to firms listed in the maritime cluster database maintained by the University of Turku (cf. Karvonen et al. 2016). The first reminder message was sent 4 weeks after the survey was opened, and the second one 2 weeks later. In total, 226 usable responses were received, of which 188 were from the maritime industry (105) and maritime logistics (83), giving total response rate of 15 and 13.4%, respectively: this is well in line with other large-scale surveys in the field (Wagner and Kemmerling 2010).

Non-response bias was tested by comparing the early and late respondents (Armstrong and Overton 1977). The early respondents replied during the first 4 weeks before the first reminder mail was sent. A *t*-test of difference was conducted on the mean responses to the current and future significance of the competence variables. Statistically significant differences were identified at *p* < 0.05 in one of the 32 variables (project competence), which indicates a low risk of non-response bias.

The survey respondents were asked to assess a list of competences (Table 1) from two perspectives. First, they were asked to estimate the importance of the competences for their organization currently and after 5 and 10 years on a 5-point Likert scale ranging from 1 no to 5 critical significance. Second, they were asked to evaluate the current levels of the same set of competences in their organization, the preferred current levels, and the preferred levels in 5 years. Competence level was assessed on a 7-point Likert scale (4…10). Separate estimates were given for occupations requiring vocational and university education.

**Table 1:** Categorization of competences

Competence	LeDeist and Winterton 2005	Tatham et al. 2017	Tassabehji and Moorhouse 2008	Kotzab et al. 2018
Risk management (incl. security)	Cognitive	PSS	SB	Analytic
Environmental regulations and technology	Cognitive	PSS	SB	Analytic
Occupational safety	Functional	FS	TE	Basic
Occupational learning	Meta	GMS	IP	Learning
Formal qualifications	Cognitive	FS	TE	Learning
Problem solving and innovativeness	Meta	PSS	SB	Analytic
Production methods and automation	Functional	FS	TE	Basic
Project competence	Cognitive	GMS	TE	Specific
Operations in large networks	Meta	GMS	EE	Analytic
Language skills	Functional	FS	TE	Specific
Teamwork	Social	IPS	IP	Social
Competence management	Meta	GMS	IP	Analytic
Ethicalness and responsibility	Cognitive	PSS	SB	Specific
Flexibility and change management	Social	IPS	IP	Analytic
Understanding of customers’ business	Cognitive	GMS	SB	Specific
Quality management	Cognitive	PSS	IE	Analytic

Estimates of competences were based on previous studies of competence in the maritime and logistics fields (see Appendix). These studies used a total of 93 competence items, of which 32 competences were selected for use in the survey. Some of these were industry-specific (the maritime industry or maritime logistics). For the purposes of this research, only the 16 competences that seemed to suit both industries were used. Of the 16 chosen ones, it could be argued that formal qualification is not a competence as such. However, qualification is defined as “an official record showing that you have finished a training course or have the necessary skills” (Cambridge Dictionary 2020). Therefore, including it in the survey works as a comparison to whether the formal qualification is considered to meet the emerging competence needs.

The selected competences have previously been classified in different ways, as Table 1 shows. LeDeist and Winterton (2005) refer to cognitive, functional, social, and meta-competences, whereas Kotzab et al. (2018) distinguish between basic, analytic, learning, and specific competences. Tatham et al. (2017) and Tassabehji and Moorhouse (2008), in turn, have their own classifications. It thus seems that the categorization is indicative of and depends on the context. Risk management, for example, could be strictly rule-based and thus functional, or it could be more conceptual and cognitive in approach.

The questionnaire setup described above allowed us to construct a set of variables. First, we compare the importance of competences currently and after 5 years to get an idea of where the biggest changes are likely to occur. Second, we follow the example of Lorentz et al. (2013) and calculate a so-called competence gap, which indicates the difference between the real and desired levels of competence. We calculated this variable separately for the current situation and for the estimated situation in 5 years.

## Results

**Fig. 1:**
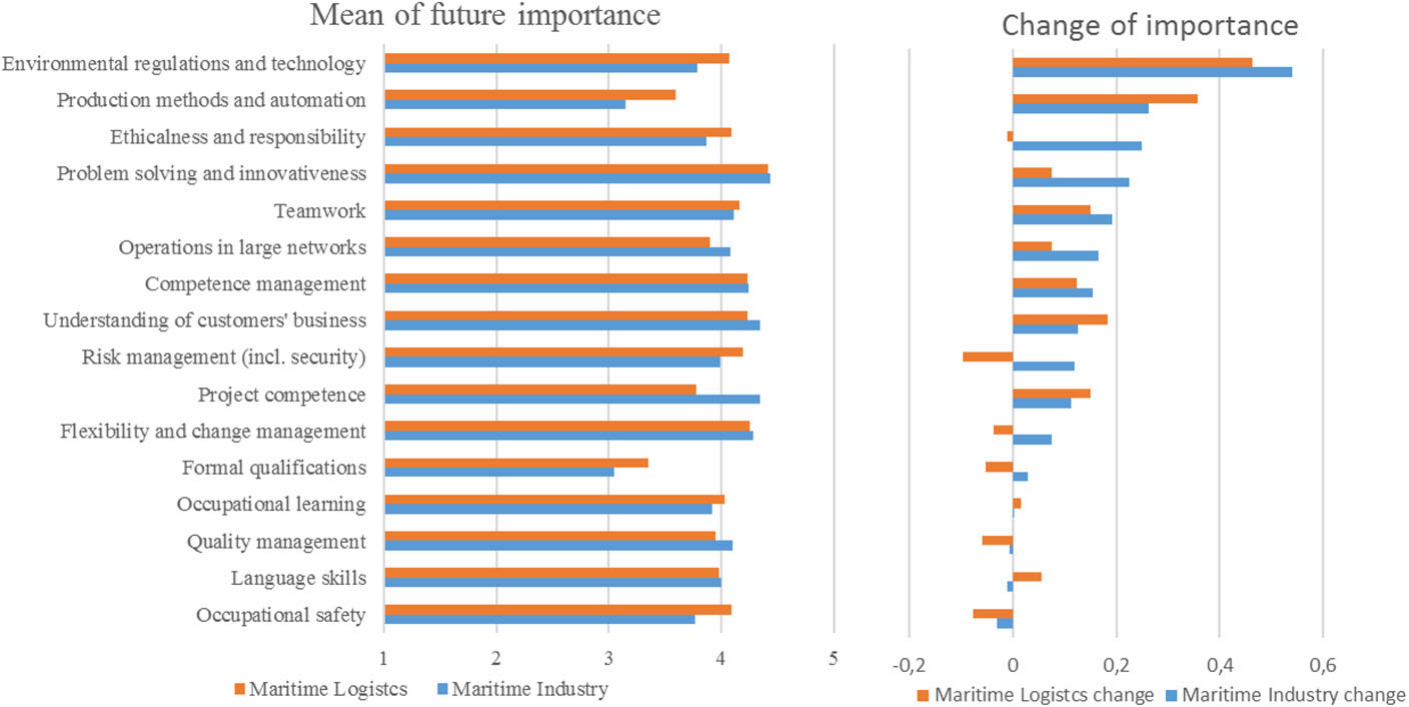
The future importance of competences in maritime logistics and the maritime industry

Figure 1 illustrates the future importance of competences (left chart) and the expected change in the importance in 5 years (right chart). The results indicate that the belief in both sectors is that competences such as problem solving and innovativeness, flexibility, and both change and competence management will be the most highly valued in the future. The maritime industry also considers project competence highly important. In both sectors, the least importance was attached to production methods and automation as well as formal qualifications, both currently and in 5 years.

Our first proposition was that maritime logistics and the maritime industry share common view on importance of the new competences, which largely turned out to be the case. It is clear from the figure that, both currently and for the future, workplace learning, problem solving, quality, customer business, networking, language skills, competence management, and flexibility are all assessed similarly in the two sectors. Business ethics, risk management, and environmental technology are more critical for maritime logistics, but the only statistically significant differences between the two sectors concerned production methods and project competence (*p*<.05).

Our second and third propositions were that institutional pressure would widen the skills gap in environmental technology (P2) and automation (P3) in the maritime cluster. Figure 1 also shows the difference between the current state and the future view, the biggest change clearly being in “environmental regulations and technology” and “production methods and automation.” Overall, the perceived importance of practically all of the competences under investigation will increase in the future.

**Fig. 2:**
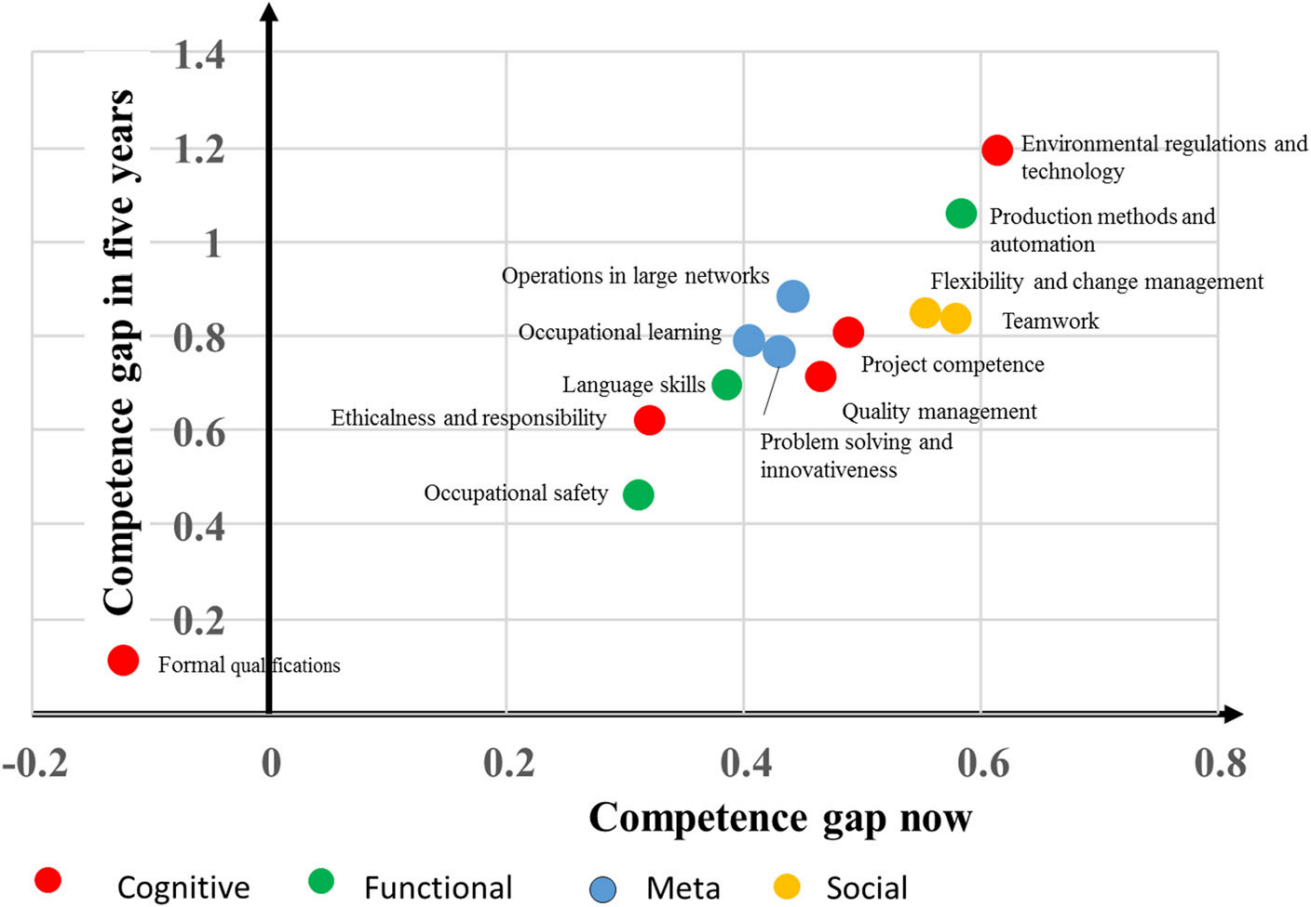
Competence gap (required level–current level) now and 5 years hence in vocational-level maritime employment

**Fig. 3:**
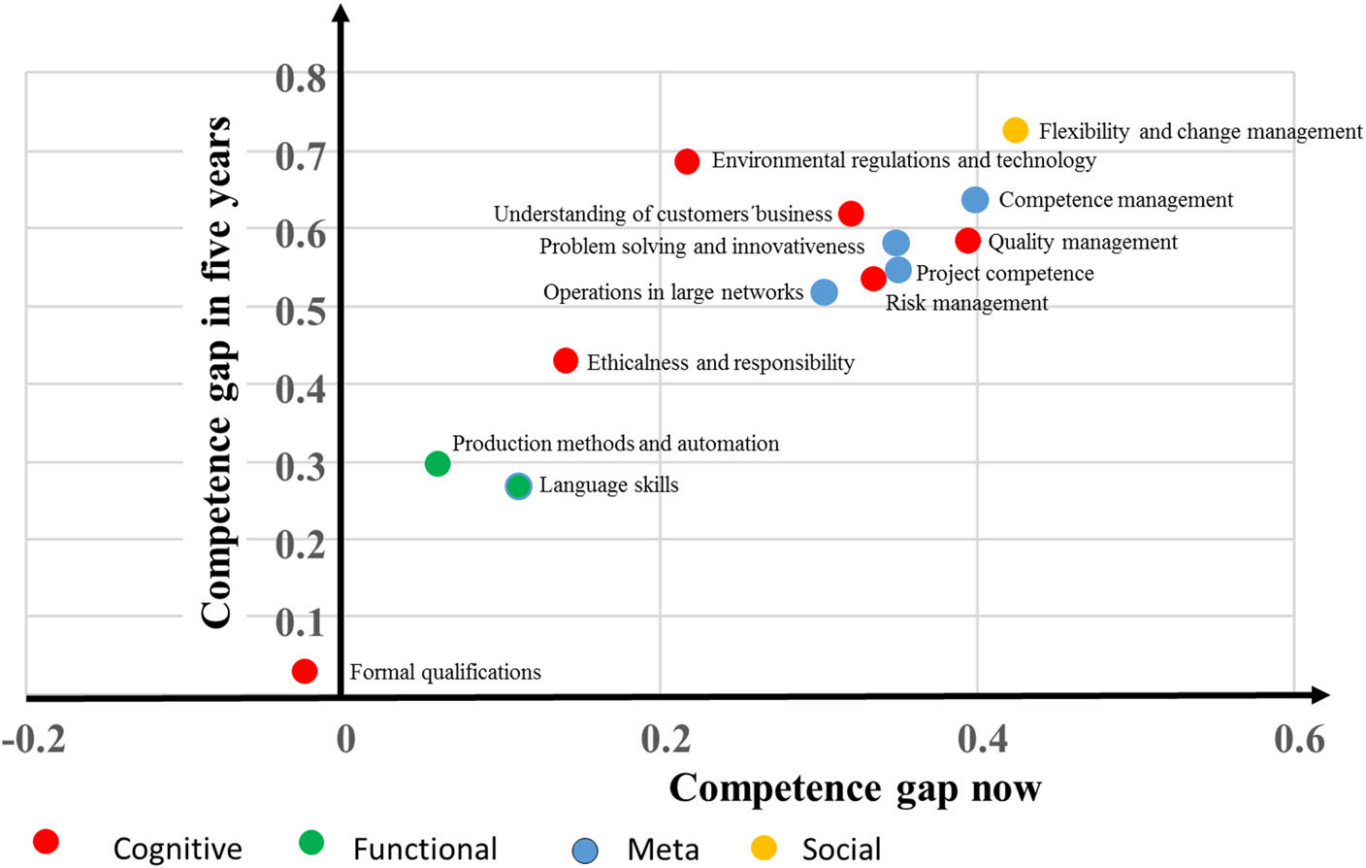
Competence gap (required level–current level) now and 5 years hence in university-level maritime employment

Figures 2 and 3 present the competence gaps (required vs the currently achieved level of competence) in vocational- and university-level maritime employment. It would seem that the widest competence gap on the vocational level, both currently (0.61) and in 5 years (1.19), is in environmental regulations and technology, followed by production methods and automation (0.58 and 1.06), teamwork (0.58 and 0.83), and flexibility and change management (0.55 and 0.84). Two further observations can be made. First, there appears to be a positive competence gap in all areas apart from formal qualifications, in which the current gap is negative. This would indicate that, currently, there is even an oversupply of formally qualified people, although this is expected to turn into a deficit 5 years hence. Second, the gap is expected be wider in all competence areas 5 years hence. All in all, the results imply that even currently there is a need for a more competent workforce and that this is expected to be even more acute in the future.

Figure 3 presents the same results for university-level maritime employment. Overall, the competence gaps both now and in the future are narrower than on the vocational level. There are also some differences in the widest gaps: on the university level, the widest gap was in flexibility and change management, both currently (0.42) and in the future (0.72), followed currently by competence management (0.4) and quality management (0.39).

As on the vocational level, among the widest competence gaps (0.68) in university-level employment, 5 years hence will be in environmental regulations and technology. The narrowest gaps on the university level both now and in 5 years will concern production methods and automation, and language skills, although a widening is expected in the future. Just as on the vocational level, the gap in formal qualifications is currently negative (−0.02), and is expected only to become barely positive (0.02) in 5 years.

**Fig. 4:**
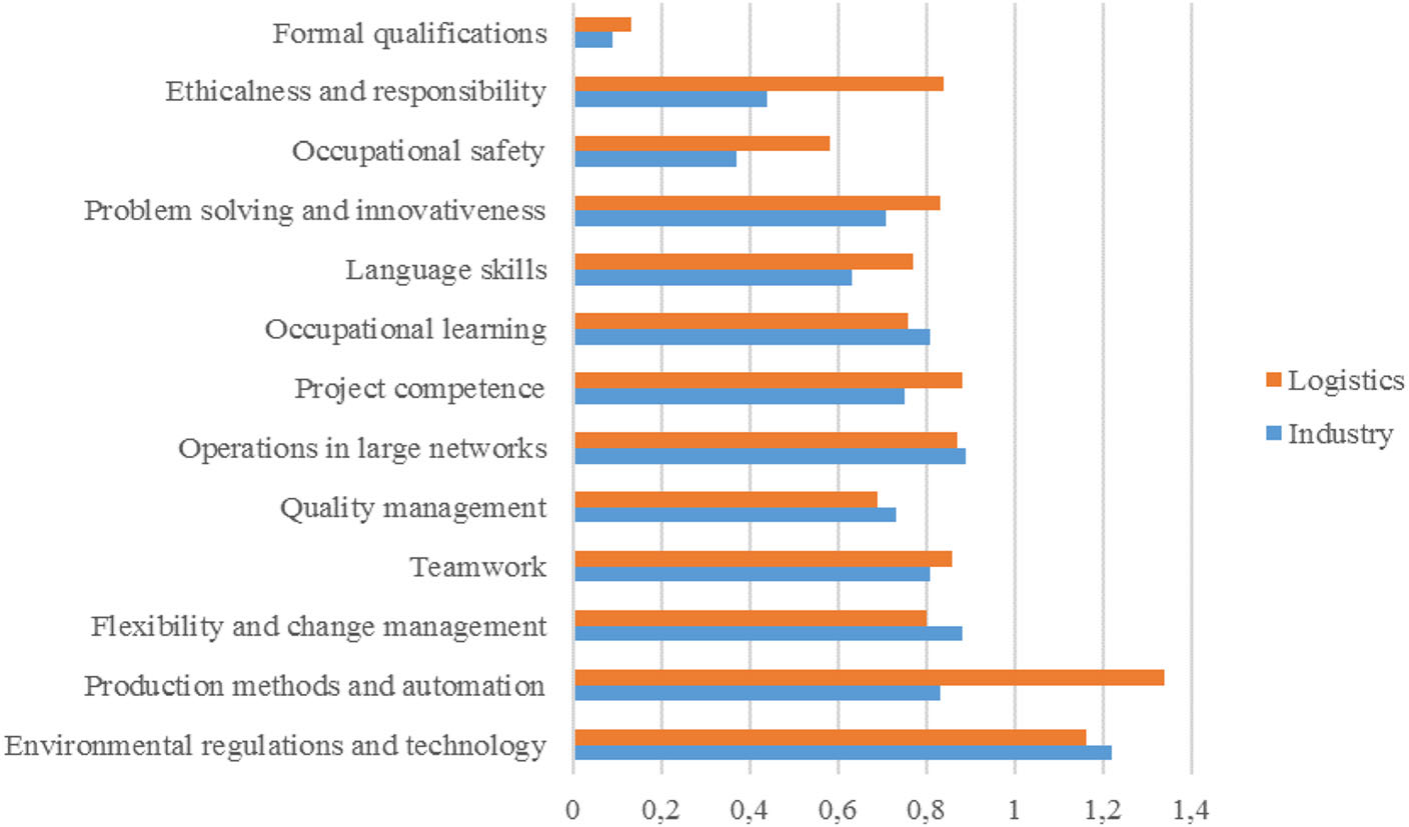
A comparison of future competence gaps in vocational-level employment

**Fig. 5:**
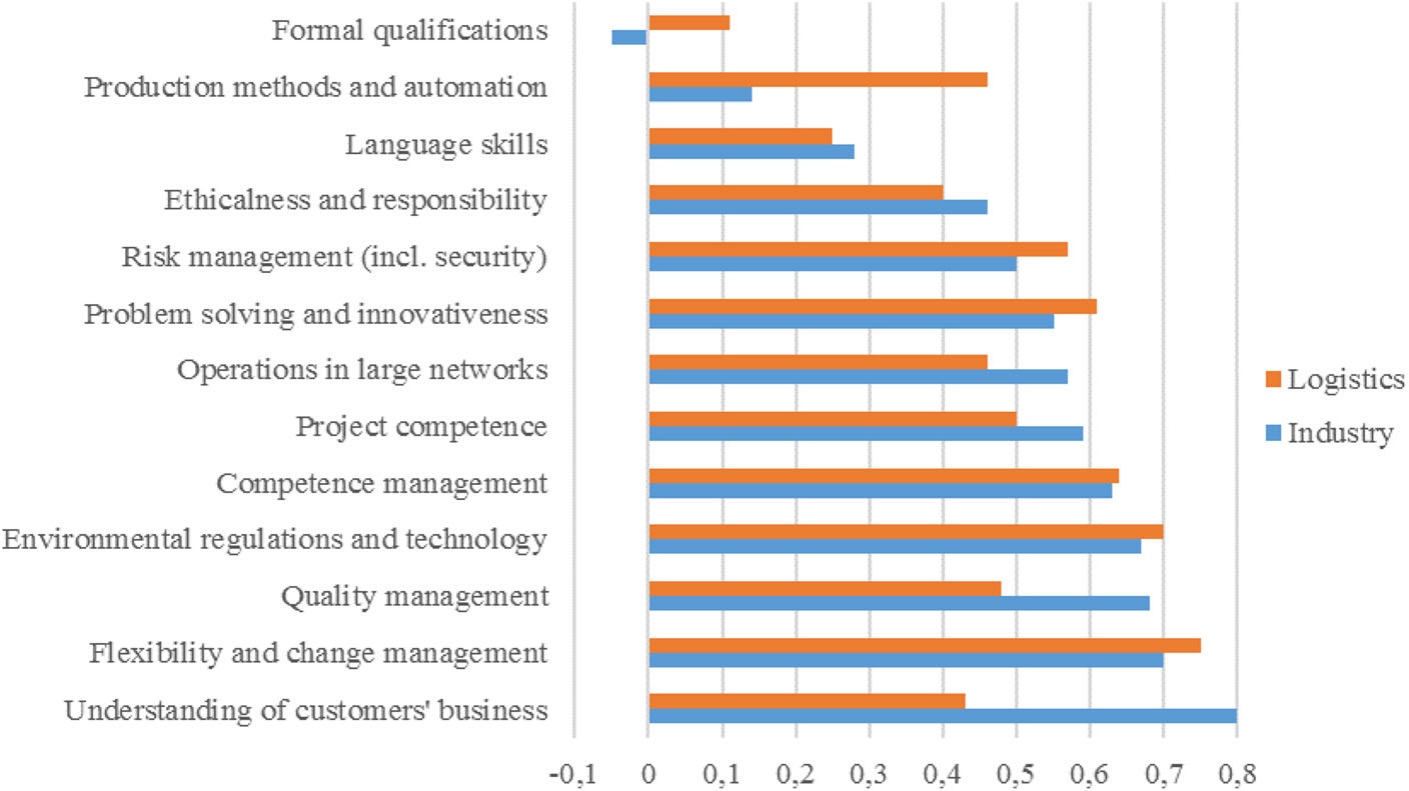
A comparison of future competence gaps in university-level employment

Our fourth proposition was that, as a cluster, maritime logistics and the maritime industry share a common vision on competence gaps in the future. Figures 4 and 5 show the differences between the two. As we posited, especially on the vocational level, the opinions are very similar. The only large differences concern production methods and automation, and ethicalness and responsibility, the estimated future competence gap being significantly higher in maritime logistics than in the maritime industry. On the university level, however, the results are less consistent. Primarily, it would seem that the competence gaps are wider in the latter than in the former. The largest differences between the two concern the understanding of customers’ business and quality management.

Our fifth proposition was that, in the future, the gap in cognitive and meta-competences will be wider than in functional and operational skills. This proposition is supported, especially with regard to university-level employment (see Fig. 3), as the two competences with the smallest gaps (excluding formal training) concern functional skills. In the case of vocational employment (Fig. 2), however, the competence gaps vary in the different categories.

## Discussion and conclusions

Environmental regulations and development of required technologies are rapidly changing the operating environment of maritime industry. The aim of this paper is to present empirical evidence on how the competence needs of the maritime industry and maritime logistics are evolving, presumably due to these changes. To do this, we analyzed survey data collected from maritime logistics firms such as shipping companies and port operators and maritime-industry players such as shipyards and equipment manufacturers. We compared gaps, first, between the current and the desired skills levels, and second, between the current and future importance of competences.

The World Economic Forum report (WEF 2016) urges organizations to prepare for a fourth industrial revolution driven by technological advancements in a wide spectrum of jobs. The relative need for cognitive abilities, problem solving, and system-related skills will increase more rapidly than for technical skills and physical abilities. Our results reflect this, but it seems that rapid revolution will be followed by a continuous learning process.

Our findings show that firms in the maritime industry and in maritime logistics have similar views on the current and future importance of different competences. This result supports the assumption that, at least from a competence perspective, the two sectors form an industrial cluster as defined by Delgado et al. (2014) and Rosenfeld (2005). Both consider social and meta-competences such as problem solving, flexibility, and competence management the most important. As such, this is not a surprise, given that similar results have been reported previously in the supply chain context (see, e.g., Kotzab et al. 2018). Similarly, both sectors consider formal qualifications less important. This result gives an interesting insight: in practice, the firms in both sectors are less interested in the formal education of the employee and place more value on continuous learning and skills and competences that could be considered less task-specific or functional.

The second finding is associated with future developments. The two competences that will increase most in importance in both sectors concern environmental regulations and technology and production methods and automation. Both of these could be associated with ongoing major changes in the maritime sector, as Cicek et al. (2019) suggest. The shipping business has already faced a series of new environmental regulations, and tighter restrictions both in the IMO and the EU are likely to follow. As institutional theorists suggest, this coercive pressure (Zucker 1987) obviously affects the need for competences. Similarly, technological developments (Hinings et al. 2018), even in the form of autonomous vessels, are constantly increasing the role of automation in shipping and shipbuilding. Naturally, this new norm is also visible in the workforce requirements set for the sector. According to Amdam and Bjarnar (2015), a resilient cluster is adaptive to globalization shock and is able to establish a dynamic link from customer demand to supply. A dynamic cluster develops its specialization in a competitive environment, generating new know-how and innovations when it has access to skilled labor and an appropriate infrastructure (Stavroulakis and Papadimitriou 2016).

The survey setup also enabled the calculation of the so-called competence gap between the current and the desired level, both currently and after 5 years. It would seem that, overall, the gap is wider on the vocational level than on the university level and is likely to increase. At the same time, neither of the sectors considers formal qualifications a major problem nor expects much increase in the desired level of formal education. This, as such, is an interesting finding, implying that firms in the industry see the need for a more competent workforce, but do not think the solution lies in formal education. The nature of the competences with the widest gaps differs on the two employment levels. It would seem that there are as wide gaps in functional competences as in cognitive, meta-, and social competences on the vocational level, whereas the widest gaps on the university level are in social and meta-competences and the narrowest ones (excluding formal qualifications) in functional competences. This is interesting given the nature of university education, the main aim of which is to develop cognitive and meta-competences rather than functional skills. On the other hand, the finding of expected future gaps in cognitive and meta-competences in vocational training, too, are well in line with previous assumptions put forward by Manuel (2017) about a transition towards more general instead of task-dependent competences.

Our findings apply the institutional perspective lens to assess of maritime education needs. This research contributes on several levels. First, we are able to identify a joint need for competences in the maritime industry and in maritime logistics, which thereby form a maritime cluster. Second, we recognize both current and future competence needs in this cluster. We hope these results will be of use both to policymakers in developing the existing curriculum to better meet future needs and to firms in the cluster in preparing their competence base for years to come.

Our research is by no means without limitations. Even if our sample is of a considerable size, it is still limited to companies in a single country. At the same time, the companies are not exclusively Finnish; in fact, most of them are international and some are even global leaders in their respective industries. Therefore, their perspectives on competence needs are not limited to Finland, or to Finnish conditions, and could also be considered valid for international stakeholders. Naturally, the results would be enhanced by further research involving respondents from firms with a different geographical scope.

## Data Availability

Not applicable

## Appendix. Previous studies about maritime and logistics competences

Anttila, R., & Salmenhaara, T. (2011) Merenkulkualan koulutuksen tila ja kehittämistarpeet. Finnish National Agency for Education .http://www.oph.fi/download/131319_Merenkulkualan_koulutuksen_tila_ja_kehittamistarpeet.PDF

APICS (2014) Supply Chain Manager Competency Model. The Association for Supply Chain Management. http://www.apics.org/*APCS*.

Ellinger, A. E., & Ellinger, A. D. (2014) Leveraging human resource development expertise to improve supply chain managers’ skills and competencies. European Journal of Training and Development, *38*(1–2), 118–135.

EU (2009a) Building and Repairing of Ships; Comprehensive sectoral analysis of emerging competences. European Commission.

EU (2009b) Transport and logistics; Comprehensive sectoral analysis of emerging competences. European Commission.

Fjeld, G. P., Tvedt, S. D., & Oltedal, H. (2018) Bridge officers’ non-technical skills: a literature review. WMU Journal of Maritime Affairs, (1), 475–495.

Gammelgaard, B., & Larson, P. D. (2001) Logistics Skills and Competencies for Supply Chain Management. Journal of Business Logistics, 22(2), 27–50.

Giunipero, L., Handfield, R. B., & Eltantawy, R. (2006) Supply management’s evolution: key skill sets for the supply manager of the future. International Journal of Operations & Production Management, *26*(7), 822–844.

Jordan, C., & Bak, O. (2016) The growing scale and scope of the supply chain: a reflection on supply chain graduate skills. Supply Chain Management, *21*(5), 610–626.

Klumpp, M. (2018) Automation and artificial intelligence in business logistics systems: human reactions and collaboration requirements. International Journal of Logistics Research and Applications, 21(3), 224–242

Kotzab, H., Teller, C., Bourlakis, M., & Wünsche, S. (2018) Key competences of logistics and SCM professionals – the lifelong learning perspective. Supply Chain Management, 23(1), 50–64.

Lorentz, H., Töyli, J., Solakivi, T., & Ojala, L. (2013) Priorities and determinants for supply chain management skills development in manufacturing firms. Supply Chain Management: An International Journal, 18(4), 358–375.

LVM. (2014) *Suomen meriliikennestrategia 2014-2022*. Retrieved from http://urn.fi/URN:ISBN:978-952-243-388-6

OPH (2012) Meriteollisuuden Osaamistarpeet 2025. Finnish National Agency for Education

OPH (2018) Liikenne- ja Logistiikka-alan Osaamis- ja koulutustarpeiden kehitysnäkymiä. Finnish National Agency for Education

Oravasaari, T., Paavo, J., & Nissilä, J. (2015) *Mahdollisuuksien meri – 23 suositusta Suomen meriklusterin osaamisen kehittämiseksi*. KYAMK_B147.pdf

Solakivi, T., Ojala, L., Töyli, J., Hälinen, H.-M., Lorentz, H., Rantasila, K., Laari, S. (2010) *Logistiikkaselvitys*. Ministry of Transport and Communications of Finland

TEM. (2017) Smart Maritime Technology Solutions. Ministry of Economic Affairs and Employment of Finland

Thai, V. V. (2012) Competency requirements for professionals in logistics and supply chain management. *International Journal of Logistics Research and Applications*, *15*(2), 109–126. 10.1080/13675567.2012.694859

Vaahtera, A., Sepponen, S., Larvus, L., & Hjelt, M. (2016) *Klusteriennakoinnin esiselvitys*. Gaia Consulting

## References

[CR1] Amdam RP, Bjarnar O (2015). Globalization and the development of industrial clusters: comparing two Norwegian clusters, 1900-2010. Bus Hist Rev.

[CR2] Armstrong JS, Overton TS (1977). Estimating nonresponse bias in mail surveys. J Mar Res.

[CR3] Bals L, Schulze H, Kelly S, Stek K (2019). Purchasing and supply management (PSM) competencies: current and future requirements. J Purch Supply Manag.

[CR4] Beer J, Meethan K (2007). Maritime and maritime sector skills shortages in the South West of England: developing regional training provision. J Vocat Educ Training.

[CR5] Bessen J (2016) How computer automation affects occupations: technology, jobs and skills, Boston University School of Law. Law & Economics Working Paper No. 15-40

[CR6] Birkinshaw J, Gibson C (2004). Building ambidexterity into an organization. MIT Sloan Manag Rev.

[CR7] Caesar LD, Cahoon S, Fei J, Sallah CA (2020). Exploring the antecedents of high mobility among ship officers: empirical evidence from Australia. Marit Policy Manag.

[CR8] Cambridge Dictionary (2020) https://dictionary.cambridge.org/. Accessed 27 Dec 2020

[CR9] Christensen CM (2006). The ongoing process of building a theory of disruption. J Prod Innov Manag.

[CR10] Christopher M, Holweg M (2011). “Supply Chain 2.0”: Managing supply chains in the era of turbulence. Int J Phys Distr Log.

[CR11] Cicek K, Akyuz E, Celik M (2019). Future skills requirements analysis in maritime industry. Procedia Comput Sci.

[CR12] Cohen WM, Levinthal DA (1990). Absorptive capacity: a new perspective on learning and innovation. Adm Sci Q.

[CR13] Delgado M, Porter ME, Stern S (2014). Clusters, convergence, and economic performance. Res Policy.

[CR14] Dimaggio PJ, Powell WW (1983). The Iron Cage Revisited: Institutional Isomorphism and Collective Rationality in Organizational Fields. Am Sociol Rev.

[CR15] Dinwoodie J (2000). The perceived importance of employment considerations in the decisions of students to enroll on undergraduate courses in Maritime Business in Britain. Marit Policy Manag.

[CR16] Djoumessi A, Chen SL, Cahoon S (2019). Factors influencing innovation in maritime clusters: an empirical study from Australia. Mar Policy.

[CR17] Doloreux D (2006). The Quebec’s coastal maritime cluster : innovative and locally embedded?. J Small Bus Entrepr.

[CR18] Dooley K (1997). A complex adaptive systems model of organization change. Nonlin Dynam Psychol.

[CR19] Engeström Y (2007). From stabilization knowledge to possibility knowledge in organizational learning. Manag Learn.

[CR20] Ferritto VR (2016). Maritime education factors and presenteeism: a comparative quantitative study. WMU J Marit Aff.

[CR21] Fjeld GP, Tvedt SD, Oltedal H (2018). Bridge officers’ non-technical skills: a literature review. WMU J Marit Aff.

[CR22] Flatten TC, Engelen A, Zahra SA, Brettel M (2011). A measure of absorptive capacity: scale development and validation. Eur Manag J.

[CR23] Francalanci C, Morabito V (2008). IS integration and business performance: the mediation effect of organizational absorptive capacity in SMEs. J Inf Technol.

[CR24] Gardner B, Naim M, Obando-Rojas B, Pettit S (2001). Maintaining the maritime skills base: does the Government have a realistic strategy?. Marit Policy Manag.

[CR25] Gekara V (2009). Understanding attrition in UK maritime education and training. Glob Soc Educ.

[CR26] Green F (2012). Employee involvement, technology and evolution in job skills: a task-based analysis. Ind Labor Relat Rev.

[CR27] Hammervoll T, Halse LL, Engelseth P (2014). The role of clusters in global maritime value. Int J Phys Distr Log.

[CR28] Hinings B, Gegenhuber T, Greenwood R (2018). Digital innovation and transformation: an institutional perspective. Inf Organ.

[CR29] ICS (2020) Shipping and World Trade: Global Supply and Demand for Seafarers, Retrieved from https://www.ics-shipping.org/shipping-facts/shipping-and-world-trade/global-supply-and-demand-for-seafarers. Accessed 14 May 2020

[CR30] International Association of Maritime Universities (2019) Global Maritime Professional, Body of Knowledge 2019

[CR31] James AJ, Schriever UG, Jahangiri S, Girgin SC (2018). Improving maritime English competence as the cornerstone of safety at sea: a focus on teaching practices to improve maritime communication. WMU J Marit Aff.

[CR32] Johns M (2018). Seafarers and Digital Disruption.

[CR33] Karvonen T et al. (2016) Suomen meriklusteri kohti 2020 lukua. Ministry of Economic Affairs and Employment of Finland

[CR34] Klumpp M (2018). Automation and artificial intelligence in business logistics systems: human reactions and collaboration requirements. Int J Log Res Appl.

[CR35] Kotzab H, Teller C, Bourlakis M, Wünsche S (2018). Key competences of logistics and SCM professionals – the lifelong learning perspective. Supply Chain Manag.

[CR36] Laaksonen E, Mäkinen H (2013). The competitiveness of the maritime clusters in the Baltic Sea region: key challenges from the Finnish perspective. J East West Bus.

[CR37] Lau Y, Ng AKY (2015). The motivations and expectations of students pursuing maritime education. WMU J Marit Aff.

[CR38] LeDeist FD, Winterton J (2005). What is competence?. Hum Resour Dev Int.

[CR39] Lorentz H, Töyli J, Solakivi T, Ojala L (2013). Priorities and determinants for supply chain management skills development in manufacturing firms. Supply Chain Manag.

[CR40] Makkonen T, Inkinen T, Saarni J (2013). Innovation types in the Finnish maritime cluster. WMU J Marit Aff.

[CR41] Manuel ME (2017). Vocational and academic approaches to maritime education and training (MET): Trends, challenges and opportunities. WMU J Marit Aff.

[CR42] Mindykowski J (2017). Towards safety improvement: implementation and assessment of new standards of competence for Electro-Technical Officers on ships. Marit Policy Manag.

[CR43] Moller K (2010). Sense-making and agenda construction in emerging business networks - how to direct radical innovation. Ind Mark Manag.

[CR44] Ng AKY, Koo AC, Ho WCJ (2009). The motivations and added values of embarking on postgraduate professional education: evidences from the maritime industry. Transp Policy.

[CR45] Ng AKY, Koo AC, Pallis AA (2011). Professionalization of the shipping industry via postgraduate education. Ocean Coast Manag.

[CR46] Paine-Clemes B (2006). What is quality in a maritime education?. IAMU J.

[CR47] Piening EP, Salge TO (2015). Understanding the antecedents, contingencies, and performance implications of process innovation: a dynamic capabilities perspective. J Prod Innov Manag.

[CR48] Pinto H, Cruz AR, Combe C (2015). Cooperation and the emergence of maritime clusters in the Atlantic: analysis and implications of innovation and human capital for blue growth. Mar Policy.

[CR49] Rosenfeld S (2005) Industry clusters: business choice, policy outcome, or branding strategy? J New Bus Ideas Trends 3(2):4–13. http://www.jnbit.org/upload/Rosenfeld-3-2-2005.pdf

[CR50] Stavroulakis PJ, Papadimitriou S (2016). The strategic factors shaping competitiveness for maritime clusters. Res Transp Bus Manag.

[CR51] Tassabehji R, Moorhouse A (2008). The changing role of procurement: developing professional effectiveness. J Purch Supply Manag.

[CR52] Tatham P, Wu Y, Kovács G, Butcher T (2017). Supply chain management skills to sense and seize opportunities. Int J Logist Manag.

[CR53] Thai VV, Balasubramanyam L, Yeoh KKL, Norsofiana S (2013). Revisiting the seafarer shortage problem: the case of Singapore. Marit Policy Manag.

[CR54] Thornton PH, Ocasio W (2012) Institutional Logics. The SAGE Handbook of Organizational Institutionalism. 10.4135/9781849200387.n4

[CR55] UNCTAD (2019) Review of Maritime transport. https://unctad.org/system/files/official-document/rmt2019_en.pdf. Accessed 14 May 2020

[CR56] Wagner SM, Kemmerling R (2010). Handling nonresponse in logistics research. J Bus Logist.

[CR57] World Economic Forum (2016) The Future of Us. World Economic Forum, (Global Challenge Insight Report). 10.23943/princeton/9780691172811.003.0009

[CR58] World Maritime University (2019) Transport 2040: Automation, technology, Emplyment - the future of work. 10.21677/itf.20190104

[CR59] Wright AL, Zammuto RF (2013). Wielding the willow : processes of institutional change in English county cricket. Acad Manag J.

[CR60] Zucker L (1987). Institutional theories of organization. Annu Rev Sociol.

